# Correlation of IL-18 with Tryptase in Atopic Asthma and Induction of Mast Cell Accumulation by IL-18

**DOI:** 10.1155/2016/4743176

**Published:** 2016-03-16

**Authors:** Junling Wang, Huiyun Zhang, Wenjiao Zheng, Hua Xie, Hongling Yan, Xiaoping Lin, Shaoheng He

**Affiliations:** ^1^Allergy and Clinical Immunology Research Centre, The First Affiliated Hospital of Liaoning Medical University, Jinzhou, Liaoning 121001, China; ^2^The PLA Center of Respiratory and Allergic Disease Diagnosing Management, General Hospital of Shenyang Military Area Command, Shenyang 110840, China; ^3^Clinical Research Centre, The First Affiliated Hospital of Nanjing Medical University, Nanjing, Jiangsu 210029, China

## Abstract

Interleukin- (IL-) 18 and tryptase were previously reported to relate to asthma, but the correlation between these two potent proinflammatory molecules in asthma and their roles in mast cell accumulation remain uninvestigated. Using flow cytometric analysis technique and ovalbumin- (OVA-) sensitized mouse model, it was found that IL-18 and tryptase levels in the plasma of moderate and severe asthma were elevated, and they correlated well with each other. Tryptase and agonist peptides of protease activated receptor- (PAR-) 2 induced substantial quantity of IL-18 release. IL-18 and tryptase provoked mast cell accumulation in peritoneum of OVA-sensitized mice. OVA-sensitization increased number of IL-18 receptor (R)^+^ mast cells. IL-18 and tryptase induced dramatic increase in IL-18R^+^ mast cells and mean fluorescence intensity (MFI) of IL-18R on mast cells. Moreover, while IL-18 induced an increase in PAR-2^+^ mast cells in nonsensitized mice, IL-18 and tryptase provoked increases in IL-4 and thymic stromal lymphopoietin (TSLP) in the peritoneum of OVA-sensitized mice. In summary, the correlation between IL-18 and tryptase in plasma of patients with asthma indicates close interactions between them, which should be considered for development of anti-IL-18 and antitryptase therapies. Interactions between IL-18 and tryptase may contribute to mast cell recruitment in asthma.

## 1. Introduction

In recent years, IL-18 is emerging as an attractive participant involved in the pathogenesis of pulmonary inflammatory diseases [1]. IL-18 is a proinflammatory cytokine which was originally discovered as an interferon-*γ*-inducing factor. It is reported to promote the production of T helper (h)2 cytokines (e.g., IL-4, IL-5, IL-9, and IL-13) by T cells, NK cells, basophils, and mast cells and to act as a cofactor for Th2 cell development and IgE production [1], suggesting that it may be involved in the pathogenesis of allergic inflammation. Moreover, it was observed that IL-18 protein and IL-18R were strongly expressed in the lungs of fatal asthma [2], serum IL-18 levels were significantly higher in children who had asthma [3], and IL-18 variants were significantly associated with asthma severity [4], implicating that IL-18 may contribute to the development of asthma. However, correlations between IL-18 and tryptase in the plasma of asthmatic patients have not been investigated.

It has been reported that* Alternaria* extract induced rapid release of IL-18 from cultured normal human bronchial epithelial cells and directly initiated Th2 differentiation of naïve CD4^+^ T cells via a unique NF-*κ*B dependent pathway [5] and that the IL-1 family members IL-33 and IL-18 have been linked to induction of IL-13 production by mast cells and basophils [6]. These suggest that allergens may cause allergic reactions through IL-18. In addition, IL-18 has been observed to be able to induce release of IFN-*γ*, IL-13, and eotaxin in the lungs of ovalbumin-sensitized and challenged transgenic mice along with an increase in IL-13 producing CD4^+^ T cells and airway hyperresponsiveness [7]. However, little is known about the effect of IL-18 on other Th2 cytokine release.

Mast cells have long been accepted as the primary effector cells of allergy, and accumulation of mast cells in inflammatory regions is a pivotal event of allergy. It was reported that IL-29 was able to induce mast cell accumulation in the peritoneum of mice [8]. We then anticipated that IL-18 may also play a role in mast cell accumulation.

Tryptase has been recognized as a specific marker of mast cell activation and a proinflammatory mediator of allergic inflammation. For example, it can induce inflammatory cell accumulation [9] and vascular leakage [10]* in vivo* and provoke IL-13 release from P815 cells [11] and TNF-*α*, IL-6, and IL-1*β* from peripheral mononuclear cells [12]. It was observed that tryptase levels in serum [13] and bronchoalveolar lavage fluid [14] of patients with atopic asthma were elevated. APC 366, a selective inhibitor of mast cell tryptase, was found to significantly reduce the magnitude of antigen-induced late allergic reaction (LAR) in atopic asthmatics following its short-term repeated administration, which supports the role of mast cell tryptase in the pathophysiology of the LAR [15]. These observations strongly indicate that tryptase is likely a key proinflammatory mediator involved in the pathogenesis of atopic asthma.

In order to further understand the contributions of tryptase to atopic asthma we investigate the influence of tryptase on IL-18 release and activities in the current study. The aim of the current study is to investigate the correlation of IL-18 with tryptase in atopic asthma, the role of IL-18 and tryptase in mast cell accumulation and Th2 cytokine release, and interaction between IL-18 and tryptase.

## 2. Materials and Methods

### 2.1. Reagents

The following compounds were purchased from Sigma-Aldrich (St. Louis, MO, USA): Leupeptin, Aprotinin, RANTES, OVA (grade V), and trypan blue. Mouse IL-4 and TSLP enzyme-linked immunosorbent assay (ELISA) kits, FITC conjugated anti-mouse CCR3, Alexa Fluor® 647 conjugated anti-mouse CCR3, and PE-Cy7 conjugated anti-mouse HLA-DR antibodies were supplied by BioLegend (San Diego, USA); FITC conjugated anti-mouse PAR-2 antibody was from Santa Cruz (Santa Cruz, USA). Recombinant human lung *β* tryptase was from Promega (Wisconsin, USA). Aluminium hydroxide [Al(OH)_3_] gel adjuvant was from Brenntag Biosector (Frederikssund, Denmark). Human IL-18, mouse IL-18 ELISA kits, APC conjugated anti-mouse IL-18R, and recombinant mouse IL-18 were purchased from R&D Systems (Minneapolis, USA). Cytofix/Cytoperm*™* Fixation/Permeabilization Kits were obtained from BD Biosciences Pharmingen (Bedford, MA, USA). Human tryptase ELISA kit was from Cloud-Clone (Houston, USA). Allergens for skin prick tests were supplied by ALK-Abelló, Inc. (Denmark). The sequences of the active and reverse peptides of protease activated receptor- (PAR-) 2 were trans-cinnamoyl-Leu-Ile-Gly-Arg-Leu-Orn-amide (tc-LIGRLO-NH_2_) and trans-cinnamoyl-Orn-Leu-Arg-Gly-Ile-Leu-amide (tc-OLRGIL-NH_2_), Ser-Leu-Ile-Gly-Arg-Leu-NH_2_ (SLIGRL-NH_2_), and Leu-Arg-Gly-Ile-Leu-Ser-NH_2_ (LRGILS-NH_2_); PAR-2 antagonist peptide Phe-Ser-Leu-Leu-Arg-Tyr-NH_2_ (FSLLRY-NH_2_) was synthesized in CL Bio-Scientific Inc. (Xi'an, China). Most of the general-purpose chemicals such as salts and buffer components were of analytical grade.

### 2.2. Subjects and Animals

A total of 63 atopic asthma and 22 healthy control (HC) subjects were recruited in the study. Their general characteristics were summarized in Supplementary Table  1. (see Supplementary Material available online at http://dx.doi.org/10.1155/2016/4743176) The diagnosing criteria of atopic asthma conformed to the Global Initiative for Asthma [16]. All mild asthmatic patients were asked to stop antiallergy medication for at least 2 weeks prior to attending the study (those that could not stop antiallergy drugs were excluded). The recruited patients did not have any airway infection for more than one month. The written informed consent was obtained from each subject. The experimental procedures were approved by the Ethical Committee at Liaoning Medical University and General Hospital of Shenyang Military Area Command.

BALB/c male mice (18–22 g) were obtained from Vital River Laboratory Animal Technology Co., Ltd. (Beijing, China) (Certificate number 11400700056942/11400700056944/11400700056945/11400700056947). The animals were bred and reared under strict ethical conditions according to international recommendations. They were housed in the Animal Experimental Center of the First Affiliated Hospital of Liaoning Medical University in a specific pathogen-free environment with free access to standard rodent chow and water, at a constant temperature 23–28°C and relative humidity of 60–75%. The animal experiment procedures were approved by the Animal Care Committee at Liaoning Medical University.

### 2.3. Blood Collection

Immediately after admission (acute exacerbation stage), the blood from certain patients was collected. Blood from HC subject was collected in the outpatient clinic. From each individual, 10 mL of peripheral blood was taken into an EDTA containing tube before centrifugation at 450 g for 10 min. The plasma was collected and frozen at −80°C until use.

### 2.4. Mouse Peritoneal Injection

The procedure was mainly adopted from the one described previously by He et al. [9]. Briefly, tryptase with or without leupeptin and aprotinin, IL-18 in the presence or absence of IL-18BP, various concentrations of RANTES, tc-LIGRLO-NH_2_, tc-OLRGIL-NH_2_, SLIGRL-NH_2_, LRGILS-NH_2_, FSLLRY-NH_2_, or normal saline (NS) in 0.5 mL volume was injected into peritoneum of mice for 2 or 4 h. Mice were then sacrificed and their peritoneal lavages were collected and processed. Cells were resuspended for flow cytometric analysis of mast cells, and supernatant was stored at −80°C until use.

### 2.5. Mouse Sensitization and Challenge

Mice were sensitized on days 0, 7, 14, and 21 with a subcutaneous multipoint injection of 50 *μ*g OVA and 1.5 mg of Al(OH)_3_ suspended in NS to a total volume of 0.5 mL. Nonsensitized control animals received only the equal volume (0.5 mL) of NS on the same days. On day 25, sensitized mice were challenged with intraperitoneal injection of 0.5 mL of various concentrations of IL-18 with or without IL-18BP and tryptase with or without FSLLRY-NH_2_. At 2 or 4 h following injection, animals were killed and their peritoneal lavages were collected for analysis.

### 2.6. Flow Cytometric Analysis of IL-18R and PAR-2 Expression on Mast Cells

To detect IL-18R and PAR-2 expression on mast cells in the peritoneal lavage of mice, peritoneal cells were fixed by using Cytofix/Cytoperm kit according to the manufacturer's instructions. APC conjugated anti-mouse IL-18R, FITC conjugated anti-mouse CCR3, and PE-Cy7 conjugated anti-mouse HLA-DR or FITC conjugated anti-mouse PAR-2, Alexa Fluor 647 conjugated anti-mouse CCR3, and PE-Cy7 conjugated anti-mouse HLA-DR antibodies were added, respectively, for 30 min at 4°C. After washing, cells were resuspended in fluorescence-activated cell sorting- (FACS-) flow solution and analyzed with FACSVerse flow cytometer (BD Biosciences, San Jose, CA). A total of 50,000 events were analyzed for each sample. Data were analyzed with CellQuest software (BD Immunocytometry Systems, USA).

### 2.7. Determination of Levels of Cytokines and Tryptase

Levels of human IL-18 and tryptase, mouse IL-18, IL-4, and TSLP were measured by using ELISA kits according to the manufacturer's instruction.

### 2.8. Statistics

Statistical analyses were performed by using SPSS software (version 17.0, IBM Corporation). Data are displayed as a boxplot, which indicates the median, interquartile range, and the highest and lowest values for the number of experiments indicated. Serum levels of IL-18 or tryptase are presented as scatter plot. Whereas Kruskal-Wallis analysis indicated significant differences between groups, for the preplanned comparisons of interest, the paired Mann-Whitney *U* test was employed. For all analyses, *P* < 0.05 was considered statistically significant.

## 3. Results

### 3.1. Elevated Levels of IL-18 and Tryptase in Asthmatic Plasma

**Figure 1:**
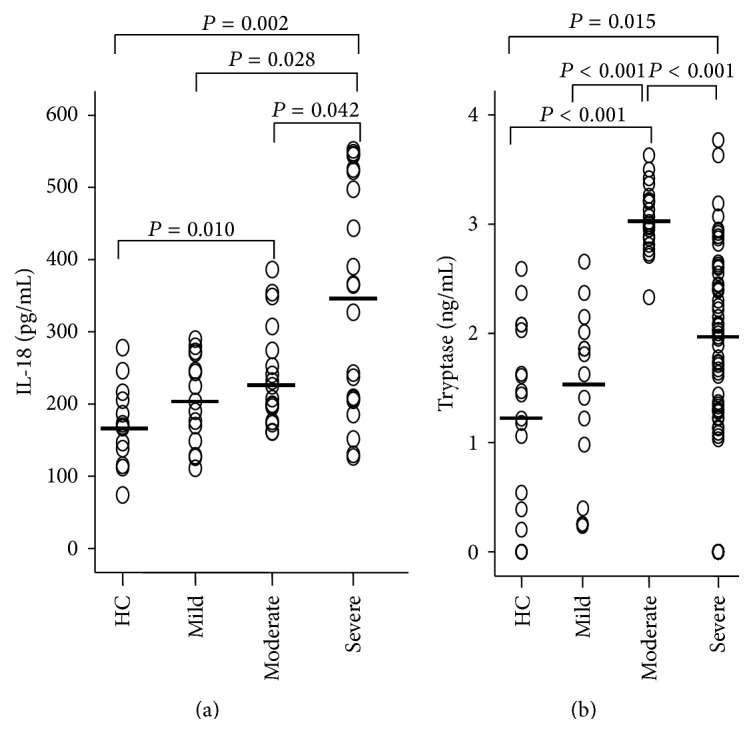
Scatter plots of levels of (a) IL-18 and (b) tryptase in the plasma of asthma. Each symbol represents the value from 1 subject. The median value is indicated with a horizontal line.

It was observed previously that IL-18 protein and IL-18 receptor were strongly expressed in the lungs of fatal asthma [2], but the correlation between IL-18 and tryptase has not been investigated before. Using ELISA kits, we observed that IL-18 levels in the plasma of moderate and severe but not mild asthma were elevated in comparison with HC subjects (Figure 1(a)). Similarly, tryptase levels in the plasma of moderate and severe asthma were higher than that of HC subjects (Figure 1(b)). It was noticed that IL-18 significantly correlated with tryptase in asthma (Tables 1 and 2).

### 3.2. Induction of IL-18 Release by Tryptase and PAR-2 Agonist Peptides in the Peritoneum of Mice

**Figure 2:**
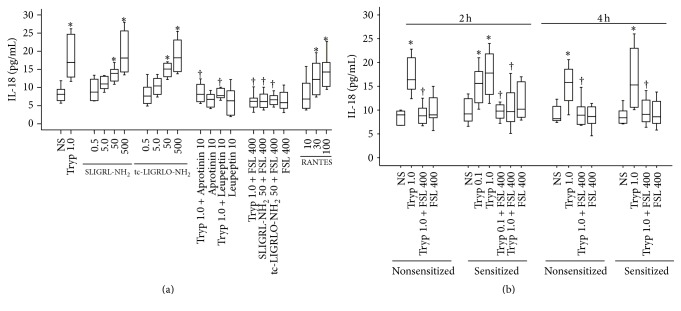
Induction of IL-18 release in the peritoneum of mice by tryptase (Tryp, *μ*g/mL) and agonists of PAR-2. (a) Nonsensitized mice were treated with Tryp with or without its inhibitors Aprotinin (*μ*g/mL) and Leupeptin (*μ*g/mL), tc-LIGRLO-NH_2_ (*μ*M), tc-OLRGIL-NH_2_ (*μ*M), SLIGRL-NH_2_ (*μ*M), LRGILS-NH_2_ (*μ*M), or FSLLRY-NH_2_ (FSL, *μ*M) for 4 h; (b) OVA-sensitized and nonsensitized mice were treated with Tryp with or without FSL for 2 or 4 h. ^*∗*^ *P* < 0.05 compared with the corresponding normal saline (NS) group. ^†^ *P* < 0.05 compared with the corresponding stimulus alone group.

Elevated levels of plasma IL-18 and tryptase and significant correlation between IL-18 and tryptase in asthma implicated that there are certain relationships between IL-18 and tryptase. Since tryptase is exclusively released from mast cells upon degranulation and mast cells are the primary effector cells of allergy, we anticipated that elevated IL-18 level may be caused by tryptase. Using mouse peritoneum model, we found that tryptase at 1.0 *μ*g/mL and agonists of PAR-2 tc-LIGRLO-NH_2_ at 50 and 500 *μ*M and SLIGRL-NH_2_ at 50 and 500 *μ*M induced substantial quantity of IL-18 release in the peritoneum of mice at 4 h following injection (Figure 2(a)). The tryptase induced IL-18 release was inhibited by Leupeptin, Aprotinin, and a PAR-2 antagonist peptide FSLLRY-NH_2_ (Figure 2(a)), implicating that the action of tryptase is likely enzymatic activity dependent and PAR-2 mediated. The reverse peptides tc-OLRGIL-NH_2_ and LRGILS-NH_2_ at the concentrations tested had little effects on IL-18 release (data not shown). It was shown that sensitization had no significant effect on tryptase induced IL-18 secretion at 2 and 4 h following injection (Figure 2(b)).

### 3.3. Induction of Mast Cell Accumulation in the Peritoneum of Mice by IL-18 and Tryptase

**Figure 3:**
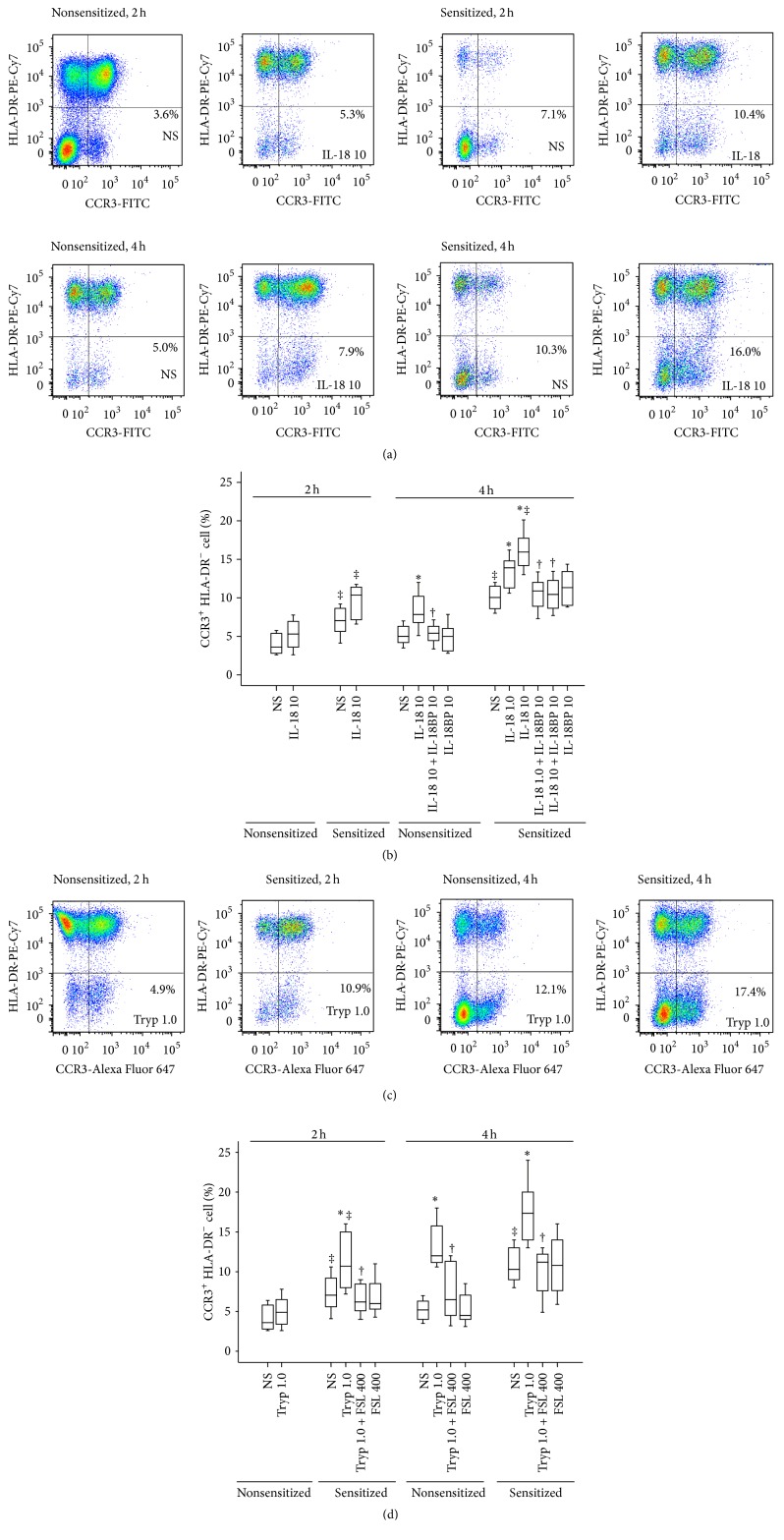
Flow cytometric analysis of IL-18 (ng/mL) and tryptase (Tryp, *μ*g/mL) induced mast cell (CCR3^+^HLA-DR^−^ cells) accumulation in the peritoneum of OVA-sensitized or nonsensitized mice. ((a) and (c)) Representative graphs of IL-18 and Tryp induced mast cell accumulation; (b) changes in the percentage of mast cells out of total peritoneal cells provoked by IL-18 in the presence or absence of IL-18BP (ng/mL); (d) changes in the percentage of mast cells provoked by Tryp in the presence or absence of FSLLRY-NH_2_ (FSL, *μ*M). ^‡^ *P* < 0.05 compared with the corresponding nonsensitized mice. ^*∗*^ *P* < 0.05 compared with the corresponding normal saline (NS) group. ^†^ *P* < 0.05 compared with the corresponding stimulus alone group.

Increased levels of tryptase implicated that the number of mast cells may be increased in asthma. We therefore examined the influence of IL-18 and tryptase on mast cell accumulation in mice. The results showed that the number of mast cells in the peritoneum of OVA-sensitized mice was 97 and 106% higher than that of nonsensitized mice (Figures 3(a) and 3(b)). It was observed that IL-18 at 10 ng/mL induced approximately up to 1.6- and 1.5-fold increase in the number of mast cells in the peritoneum of OVA-sensitized and nonsensitized mice, respectively (Figures 3(a) and 3(b)). Similarly, tryptase induced up to 1.5- and 1.4-fold increases in the number of mast cells of OVA-sensitized and nonsensitized mice at 2 h and up to 1.7- and 2.4-fold increases in the number of mast cells of OVA-sensitized and nonsensitized mice at 4 h following injection, respectively (Figures 3(c) and 3(d)). FSLLRY-NH_2_ inhibited tryptase induced mast cell accumulation when it was injected at the same time with tryptase for 2 and 4 h.

### 3.4. Induction of Upregulated Expression of IL-18R on Mouse Mast Cells by IL-18

**Figure 4:**
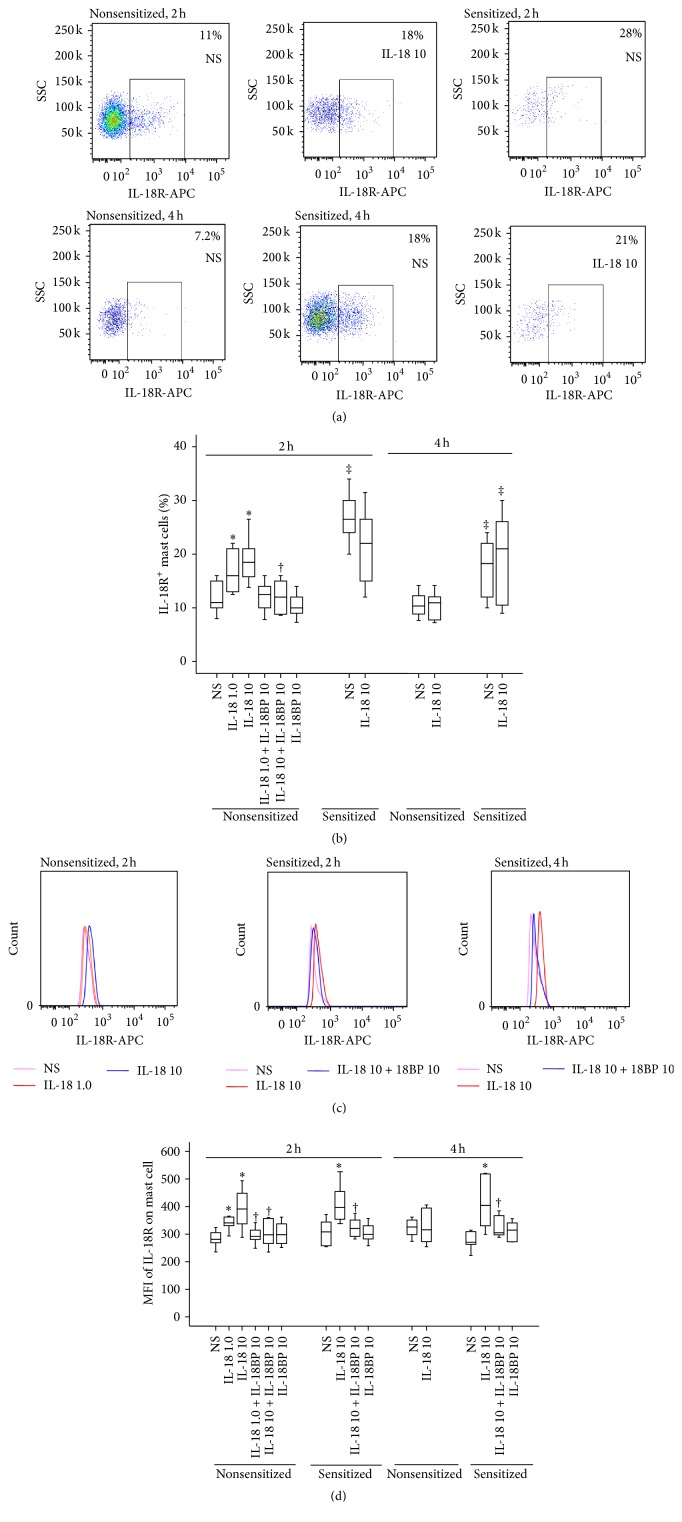
Flow cytometric analysis of IL-18 (ng/mL) induced expression of IL-18 receptor (IL-18R) on mast cells (CCR3^+^HLA-DR^−^ cells) in the peritoneum of OVA-sensitized or nonsensitized mice. ((a) and (c)) Representative graphs of the proportion of IL-18R^+^ mast cells and mean fluorescence intensity (MFI) of IL-18R on mast cell, respectively; (b) changes in the percentage of IL-18R^+^ mast cells out of total peritoneal mast cells provoked by IL-18 in the presence or absence of IL-18BP (ng/mL); (d) changes in the MFI of IL-18R on mast cell provoked by IL-18. ^‡^ *P* < 0.05 compared with the corresponding nonsensitized mice. ^*∗*^ *P* < 0.05 compared with the corresponding normal saline (NS) group. ^†^ *P* < 0.05 compared with the corresponding stimulus alone group.

To understand the potential mechanism of IL-18 induced mast cell accumulation, we examined IL-18R and PAR-2 expression on mast cells induced by IL-18. The results showed that OVA-sensitization markedly increased the number of IL-18R^+^ mast cells in the peritoneum of mice at 2 and 4 h following injection. IL-18 at 1.0 and 10 ng/mL induced 45.5 and 63.6% increases in IL-18R^+^ mast cells, respectively, in the peritoneum of nonsensitized mice at 2 h following injection. IL-18 at 10 ng/mL had little effect on the number of IL-18R^+^ mast cells at 4 h following injection (Figures 4(a) and 4(b)). However, IL-18 (10 ng/mL) elicited markedly enhanced mean fluorescence intensity (MFI) of IL-18R on mast cells from OVA-sensitized mice at 2 and 4 h following injection. At 1.0 and 10 ng/mL, IL-18 enhanced MFI of IL-18R on mast cells from nonsensitized mice by 21.1 and 39.1%, respectively, at 2 h following injection (Figures 4(c) and 4(d)). IL-18BP was able to inhibit IL-18 induced expression of IL-18R on mast cells.

### 3.5. Induction of Upregulated IL-18R Expression on Mouse Mast Cells by Tryptase

**Figure 5:**
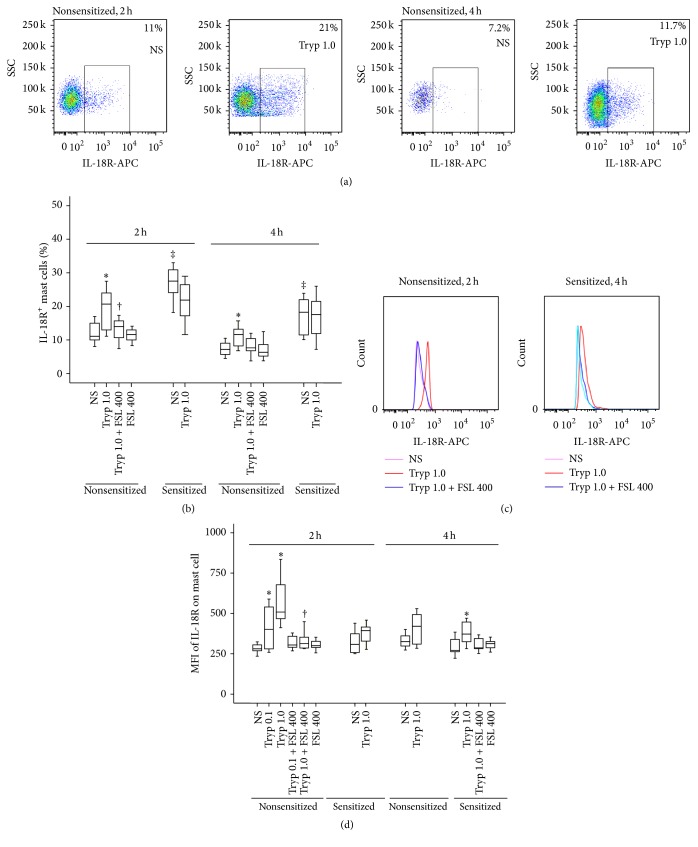
Flow cytometric analysis of tryptase (Tryp, *μ*g/mL) induced expression of IL-18 receptor (IL-18R) on mast cells (CCR3^+^HLA-DR^−^ cells) in the peritoneum of OVA-sensitized or nonsensitized mice. ((a) and (c)) Representative graphs of the proportion of IL-18R^+^ mast cells and mean fluorescence intensity (MFI) of IL-18R on mast cell, respectively; (b) changes in the percentage of IL-18R^+^ mast cells out of total peritoneal mast cells provoked by Tryp in the presence or absence of FSLLRY-NH_2_ (FSL, *μ*M); (d) changes in the MFI of IL-18R on mast cell provoked by Tryp. ^‡^ *P* < 0.05 compared with the corresponding nonsensitized mice. ^*∗*^ *P* < 0.05 compared with the corresponding normal saline (NS) group. ^†^ *P* < 0.05 compared with the corresponding stimulus alone group.

It was demonstrated previously that tryptase was able to activate mast cells via a PAR-2 dependent mechanism [17]. Therefore, we anticipated that tryptase may also affect IL-18R expression on mast cells. Using mouse peritoneum model, we showed that tryptase at 0.1 and 1.0 *μ*g/mL provoked increase in IL-18R^+^ mast cells in the peritoneum of nonsensitized but not OVA-sensitized mice at 2 and 4 h following injection (Figures 5(a) and 5(b)). Tryptase elicited also enhanced MFI of IL-18R on mast cells from nonsensitized mice at 2 h and sensitized mice at 4 h following injection (Figures 5(c) and 5(d)). Since FSLLRY-NH_2_ inhibited tryptase induced upregulation of IL-18R expression (Figures 5(b), 5(c), and 5(d)), the action of tryptase seemed PAR-2 dependent.

### 3.6. Induction of Altered PAR-2 Expression on Mouse Mast Cells by IL-18

**Figure 6:**
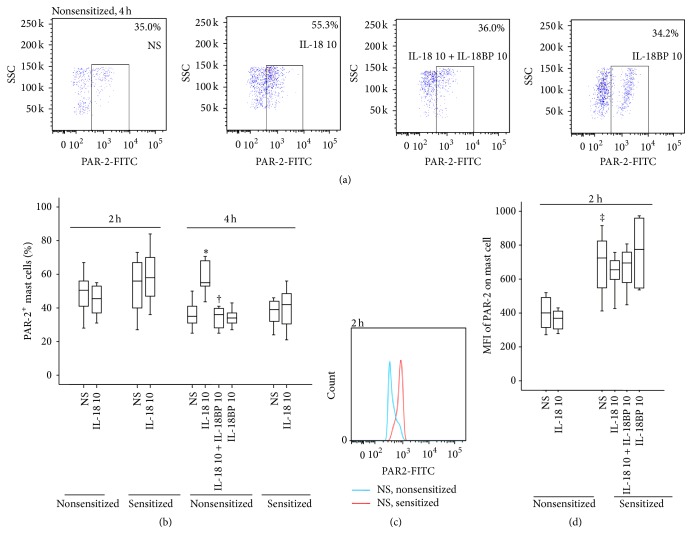
Flow cytometric analysis of IL-18 (ng/mL) induced expression of protease activated receptor- (PAR-) 2 on mast cells (CCR3^+^HLA-DR^−^ cells) in the peritoneum of OVA-sensitized or nonsensitized mice. ((a) and (c)) Representative graphs of the proportion of PAR-2^+^ mast cells and mean fluorescence intensity (MFI) of PAR-2 on mast cell, respectively; (b) changes in the percentage of PAR-2^+^ mast cells out of total peritoneal mast cells provoked by IL-18 in the presence or absence of IL-18BP (ng/mL); (d) changes in the MFI of PAR-2 on mast cell provoked by IL-18. ^‡^ *P* < 0.05 compared with the corresponding nonsensitized mice. ^*∗*^ *P* < 0.05 compared with the corresponding normal saline (NS) group. ^†^ *P* < 0.05 compared with the corresponding stimulus alone group.

To further understand the actions of IL-18 on mast cells, we investigated the influence of IL-18 on PAR-2 expression on mouse mast cells. Although sensitization had little effect on the number of PAR-2^+^ mast cells (Figures 6(a) and 6(b)), it markedly upregulated the MFI of PAR-2 expression on mast cells at 2 h following injection (Figures 6(c) and 6(d)). IL-18 at 10 ng/mL induced an increase in PAR-2^+^ mast cells of nonsensitized mice at 4 h following injection (Figures 6(a) and 6(b)).

### 3.7. Induction of Altered PAR-2 Expression on Mouse Mast Cells by Tryptase

**Figure 7:**
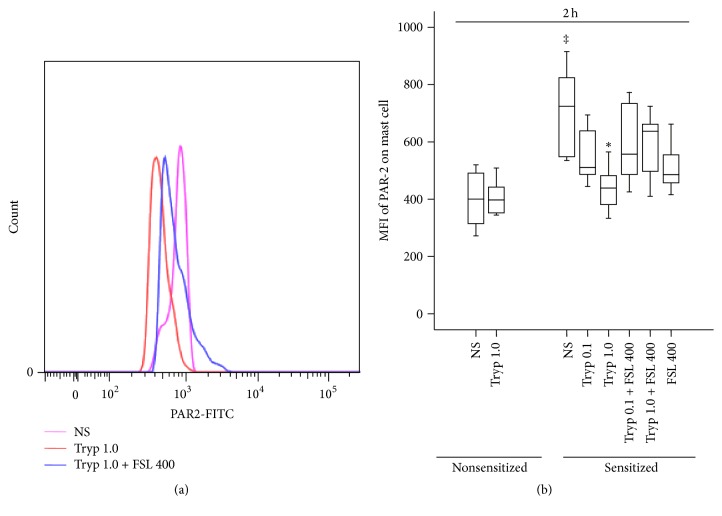
Flow cytometric analysis of tryptase (Tryp, *μ*g/mL) induced expression of protease activated receptor- (PAR-) 2 on mast cells (CCR3^+^HLA-DR^−^ cells) in the peritoneum of OVA-sensitized or nonsensitized mice. (a) A representative graph of mean fluorescence intensity (MFI) of PAR-2 on mast cell; (b) changes in the MFI of PAR-2 on mast cell provoked by Tryp. ^‡^ *P* < 0.05 compared with the corresponding nonsensitized mice. ^*∗*^ *P* < 0.05 compared with the corresponding normal saline (NS) group.

Tryptase at 0.1 and 1.0 *μ*g/mL appeared to reduce the expression of PAR-2 on mast cells from OVA-sensitized but not nonsensitized mice at 2 h following injection (Figures 7(a) and 7(b)). However, FSLLRY-NH_2_ had little effects on PAR-2 expression on mast cells.

### 3.8. Induction of Th2 Cytokine Release in the Peritoneum of Mice by IL-18 and Tryptase

**Figure 8:**
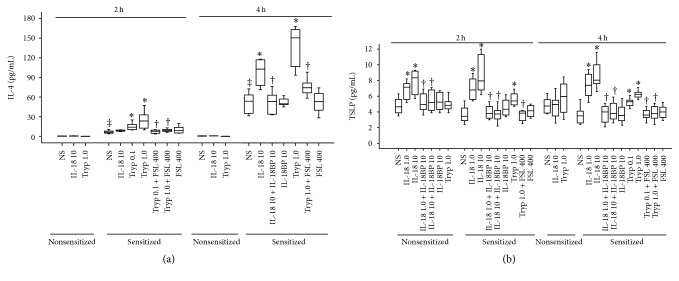
IL-18 and tryptase (Tryp) induced IL-4 (a) and thymic stromal lymphopoietin (TSLP) (b) release in the peritoneum of OVA-sensitized or nonsensitized mice. Mice were treated with IL-18 (ng/mL) in the presence or absence of IL-18BP (ng/mL), Tryp (*μ*g/mL) with or without FSLLRY-NH_2_ (FSL, *μ*M) for 2 and 4 h before their peritoneal lavage being collected for ELISA analysis. Data were displayed as a boxplot, which indicates the median, interquartile range, the largest and smallest values other than outliers (whiskers). Each piece of data represented a group of 6-7 animals. ^‡^ *P* < 0.05 compared with the corresponding nonsensitized mice. ^*∗*^ *P* < 0.05 compared with the corresponding normal saline (NS) group. ^†^ *P* < 0.05 compared with the corresponding stimulus alone group.

It has been reported previously that Th2 cytokines are involved in mast cell migration. Thus, IL-4 was identified as mast cell chemotaxin [18], and IL-10 strongly decreased RANTES-induced mast cell migration and completely inhibited mast cell migratory response to TNF [19]. TSLP is known to promote Th2 cell-associated inflammation by induction of the proliferation and differentiation of mast cells from bone marrow progenitors [20]. Since IL-18 and tryptase induced mast cell accumulation may result from increased Th2 cytokines we examined the influence of IL-18 and tryptase on Th2 cytokine release in the peritoneum of mice. The results showed that the levels of IL-4 in the peritoneum of nonsensitized mice were hardly detectable regardless of injection of IL-18, tryptase, or NS. However, OVA-sensitization dramatically enhanced the IL-4 release (up to 103.3-fold) in the peritoneum of mice. IL-18 at 10 ng/mL induced approximately 42.2 and 192% increases in IL-4 release in the peritoneum of OVA-sensitized mice at 2 and 4 h following injection. In comparison, tryptase at 1.0 *μ*g/mL provoked 3.7- and 2.8-fold increases in IL-4 in the peritoneum of OVA-sensitized mice at 2 and 4 h following injection (Figure 8(a)).

Similarly, IL-18 induced up to approximately 2.3-fold increase in TSLP release in the peritoneum of OVA-sensitized mice at 2 and 4 h following injection. In the parallel experiments, tryptase provoked up to approximately 1.8-fold increases in TSLP in the peritoneum of OVA-sensitized mice at 2 and 4 h following injection (Figure 8(b)). Moreover, IL-18 but not tryptase induced up to 1.8-fold increase in TSLP level in the nonsensitized mice at 2 h following injection (Figure 8(b)). It was noticed that IL-18 induced and tryptase-provoked cytokine release was inhibited by IL-18BP and FSLLRY-NH_2_, respectively, when they were coinjected into the peritoneum of mice.

## 4. Discussion

We have demonstrated that the plasma levels of IL-18, a proinflammatory cytokine, and tryptase, a unique mast cell degranulation marker, were elevated at admission stage of moderate and severe adult atopic asthma, confirming that these two potent proinflammatory molecules are likely to contribute to the progression of atopic asthma. Since IL-18 [4] and tryptase [21] alone have been observed to play key roles in asthma and their plasma levels correlated well in asthma, there must be some close interactions between them. Indeed, our current findings that IL-18 induced mast cell accumulation, upregulated expression of IL-18R and PAR-2 on mast cells, and Th2 cytokine release in the peritoneum of mice and that tryptase was able to provoke IL-18 release, mast cell accumulation, upregulated expression of IL-18R, and Th2 cytokine release strongly suggested that interactions between IL-18 and tryptase may play a pivotal role in atopic asthma. More importantly, our current work may provide an example that interactions between cytokines and inflammatory mediators can be more important than individual cytokine or mediator alone in the pathogenesis of allergic inflammation including atopic asthma.

It was found for the first time that tryptase was able to provoke IL-18 release in the peritoneum of mice, suggesting that this mast cell serine protease may contribute to allergic inflammation through IL-18. Since many cell types including keratinocytes, dermal macrophages, and dendritic cells are capable of producing IL-18 [22], we did not pursue the sources where IL-18 was released from in the present study. Nevertheless, the ability of tryptase in induction of IL-8 and MCP-1 release from endothelial cells [23], IL-13 release from P815 cells [11], TNF-*α*, IL-6, and IL-1*β* from peripheral mononuclear cells [12], and IL-8 release from mast cells [24] may support our observation that tryptase was able to provoke IL-18 release. While direct evidence on tryptase promoting IL-18 actions is lacking, a report that human mast cell chymase, a mast cell serine protease, rapidly cleaved recombinant pro-IL-18 at 56-phenylalanine and produced a biologically active IL-18 fragment [25] may also help to understand the action of tryptase on IL-18. It appeared that OVA-sensitization had little effect on tryptase induced IL-18 release, suggesting that the event was not related to allergen challenge in mice.

Induction of mast cell accumulation by IL-18 is an important finding as mast cells are primary effecter cells of allergy [26], and substantial quantity of this cell type is required to initiate pathological damage of the involved tissue. Cytokines that can induce mast cell accumulation or migration include the interactions of eotaxin, RANTES, and MCP-1 with CCR3 on mast cells which are responsible for the recruitment of these cells [27]; IL-6 [28] and TNF [29] stimulate migration of mast cells in the presence of laminin and SCF by itself is capable of inducing the migration of mast cells via its receptor c-Kit [30]. These reports may support our current finding that mast cell accumulation can be provoked by IL-18. The report that IL-18-dependent mastocytosis and mast cell activation are important for prompt parasite expulsion [31] may also support the current finding. OVA-sensitization seemed to enhance mast cell accumulation regardless of the presence or absence of IL-18. Induction of mast cell accumulation by OVA-sensitization was reported previously [32], which may support our current observation.

Tryptase appeared to provoke mast cell accumulation. Since tryptase is a unique product of mast cells, induction of mast cell accumulation by tryptase may represent a self-amplification mechanism, at which mast cell releases tryptase and released tryptase accumulates other mast cells. Although the action of tryptase is at least partially through activation of PAR-2, it is obvious that further work is required to confirm this self-amplification mechanism. The reports that mast cell product histamine induces chemotaxis of mouse mast cells through histamine H_4_ receptor [33] and that PAF has been identified as a potent chemoattractant of mast cells [34] may support our current observation.

IL-18 induced mast cell accumulation seemed through its ability to upregulate expression of IL-18R on mast cells as IL-18 was able to enhance expression of IL-18R on mast cells and IL-18BP was capable of blocking IL-18 induced mast cell accumulation. Despite lack of direct evidence on IL-18R mediated mast cell migration, the reports that IL-18 attracts plasmacytoid dendritic cells (DC2s) through IL-18R expression [35] and that IL-18 induced its own receptor expression and enhanced IL-18R/Nox1 binding and smooth muscle cell migration [36] may help us to understand the IL-18R mediated mast cell accumulation. OVA-sensitization appeared to enhance the number of IL-18R+ mast cells but had little effect on MFI of IL-18R on mast cells induced by IL-18 or tryptase, suggesting that the density of IL-18R on mast cells is not altered by OVA-sensitization.

Moreover, IL-18 induced upregulation of PAR-2 expression on mast cells may reinforce tryptase induced mast cell accumulation via PAR-2. While direct evidence on PAR-2 mediated mast cell accumulation is not available, the observations that mast cell tryptase induces eosinophil recruitment in the pleural cavity of mice via PAR-2 [37] and that PAR-2 induces migration of pancreatic cancer cells [38] may support our current finding. In contrast to IL-18R expression on mast cells, OVA-sensitization seemed to have little effect on the number of PAR-2^+^ mast cells but enhanced MFI of PAR-2 on mast cells, suggesting that the density of PAR-2 on mast cells can be altered by OVA-sensitization.

Induction of IL-4 and TSLP release by IL-18 in the peritoneum of mice implicates that IL-18 not only directly induces mast cell accumulation, but also indirectly causes mast cell recruitment by provoking IL-4 and TSLP release as IL-4 was reported to induce homotypic aggregation of human umbilical cord blood-derived mast cells in the presence of SCF and IL-6 [39], and TSLP was found to correlate with the number of mast cells in the conjunctival epithelium of patients with superior limbic keratoconjunctivitis [40].

As for IL-18, induction of IL-4 release by tryptase via a PAR-2 mediated mechanism implicates that tryptase may also indirectly induce mast cell accumulation by eliciting IL-4 release. The reports about induction of IL-4 release and upregulated expression of PAR-2 in P815 mast cells by GM-CSF [41] may support our view. Since TSLP is able to induce the proliferation and differentiation of mast cells from bone marrow progenitors [20] and affect mast cell growth, survival, and mediator release [42], we anticipate that this Th2 cytokine should have the ability to enhance mast cell numbers in the peritoneum of mice.

In conclusion, the correlation between IL-18 and tryptase in the plasma of the patients with atopic asthma suggested a potential connection between them. The mouse* in vivo* model used in the present study confirms that mast cells likely play a key role in mutual influence between IL-18 and tryptase. More importantly, our current work may provide an example that interactions between cytokines and inflammatory mediators can be more important than individual cytokine or mediator alone in the pathogenesis of allergic inflammation including atopic asthma, which may help to explain why some specific anticytokine or antimediator compounds are not effective in treatment of asthma.

## Supplementary Material

A total of 63 atopic asthma and 22 healthy control (HC) subjects were recruited in the study. Their general characteristics were summarized in Table S1.

## Figures and Tables

**Table 1:** Rank correlation (Spearman's correlation coefficient) between plasma levels of IL-18 and tryptase in moderate asthma.

Compound	*r* values between IL-18 and tryptase	
	IL-18	Tryptase
IL-18	1	0.925^*∗*^
Tryptase	0.925^*∗*^	1

^*∗*^ *P* < 0.05.

**Table 2:** Rank correlation (Spearman's correlation coefficient) between plasma levels of IL-18 and tryptase in severe asthma.

Compound	*r* values between IL-18 and tryptase	
	IL-18	Tryptase
IL-18	1	0.908^*∗*^
Tryptase	0.908^*∗*^	1

^*∗*^ *P* < 0.05.
