# Supplementary material for: Correlation of IL-18 with Tryptase in Atopic Asthma and Induction of Mast Cell Accumulation by IL-18
Source: Mediators Inflamm. 2016 Mar 16;2016:4743176. doi: 10.1155/2016/4743176 (PMC4812453; doi:10.1155/2016/4743176)
Supplement: Supplementary file 1 — A total of 63 atopic asthma and 22 healthy control (HC) subjects were recruited in the study. Their general characteristics were summarized in Table S1. [file 4743176.f1.doc]

**Supplement Table 1** Characteristics of adult subjects

| **Population** | **Case** | **Age (y)** | **Female/male** | **History (y)** | **Onset age (y)** |
| --- | --- | --- | --- | --- | --- |
| HC | 22 | 36 (16-65) | 14/8 | na | na |
| Asthma | 63 | 37.5 (16-61) | 37/26 | 6.0 (0.25-31) | 28 (14-53.75) |
| Allergen (+) |  |  |  |  |  |
| Mite | 21 | 38 (16-60) | 12/9 | 5.0 (0.25-15) | 31 (15-53.75) |
| Pollen | 7 | 40 (38-42) | 4/3 | 8.0 (5-22) | 34 (18-36) |
| Mite+Hair | 8 | 22 (16-50) | 5/3 | 3.0 (1-10) | 9.0 (14-40) |
| Mite+Pollen | 18 | 40 (16-61) | 10/8 | 9.5 (4-31) | 28.5 (16-51) |
| Mite+Hair+Pollen | 9 | 23.5 (16-42) | 6/3 | 5.0 (0.25-30) | 9.4 (13-41.75) |

Median values (range) are shown. Specific allergens were used for skin prick test. HC = healthy control. na = not applicable.
